# The TRPM1 channel in ON-bipolar cells is gated by both the α and the βγ subunits of the G-protein G_o_

**DOI:** 10.1038/srep20940

**Published:** 2016-02-17

**Authors:** Ying Xu, Cesare Orlandi, Yan Cao, Shengyan Yang, Chan-Il Choi, Vijayakanth Pagadala, Lutz Birnbaumer, Kirill A. Martemyanov, Noga Vardi

**Affiliations:** 1 GHM Institute of CNS Regeneration, Jinan University, Guangzhou, 510632, China; 2 Department of Neuroscience, The Scripps Research Institute, Jupiter, FL 33458, USA; 3 National Institute of Environmental Health. Sciences., Research Triangle Park, NC 27709, USA; 4 Department of Neuroscience, University of Pennsylvania, Philadelphia, PA 19104, USA; 5 Co-Innovation Center of Neuroregeneration, Nantong University, Jiangsu, China

## Abstract

Transmission from photoreceptors to ON bipolar cells in mammalian retina is mediated by a sign-inverting cascade. Upon binding glutamate, the metabotropic glutamate receptor mGluR6 activates the heterotrimeric G-protein Gα_o_β3γ13, and this leads to closure of the TRPM1 channel (melastatin). TRPM1 is thought to be constitutively open, but the mechanism that leads to its closure is unclear. We investigated this question in mouse rod bipolar cells by dialyzing reagents that modify the activity of either Gα_o_ or Gβγ and then observing their effects on the basal holding current. After opening the TRPM1 channels with light, a constitutively active mutant of Gα_o_ closed the channel, but wild-type Gα_o_ did not. After closing the channels by dark adaptation, phosducin or inactive Gα_o_ (both sequester Gβγ) opened the channel while the active mutant of Gα_o_ did not. Co-immunoprecipitation showed that TRPM1 interacts with Gβ3 and with the active and inactive forms of Gα_o_. Furthermore, bioluminescent energy transfer assays indicated that while Gα_o_ interacts with both the N- and the C- termini of TRPM1, Gβγ interacts only with the N-terminus. Our physiological and biochemical results suggest that both Gα_o_ and Gβγ bind TRPM1 channels and cooperate to close them.

In mammalian retina, an increase in light intensity hyperpolarizes the photoreceptor and initiates two opposing signals: sign-preserving synaptic transmission to the OFF bipolar cells and sign-inverting transmission to the ON bipolar cells. In darkness, the depolarized photoreceptors tonically release glutamate into the synaptic cleft, hyperpolarizing the ON bipolar cells. Light hyperpolarizes the photoreceptors, reducing glutamate in the cleft and causing the ON bipolar cells to depolarize. The key steps in this ‘sign inverting’ cascade are: glutamate activates the ON bipolar cell’s mGluR6 receptor^1^,^2^,^3^, and this activates the heterotrimeric G-protein G_o_ that comprises α_o_β3γ13^4^,^5^,^6^,^7^,^8^,^9^,^10^. Active G_o_ closes the non-selective cation channel TRPM1 (melastatin), thought to be constitutively active^11^,^12^,^13^,^14^. In the retina, TRPM1 is required for night vision as mutations in its gene or autoimmune targeting of the protein lead to lack of the ERG b-wave and to night blindness^15^,^16^,^17^,^18^. Outside the retina, two splice variants of TRPM1 regulate pigmentation in melanocytes, and loss of this gene is correlated with tumor aggressiveness in human melanoma^19^,^20^,^21^.

While there is strong evidence that active G_o_ closes the TRPM1 channel, it is not clear if this closure is caused by an active Gα_o_, or a free Gβγ dimer. Evidence indicating that Gα_o_ induces this closure is based on studies that transfected TRPM1 into CHO cells and found that applying activated Gα_o_ purified from the brain to an excised patch closed the channel, but applying Gβγ did not. These studies also found that co-transfecting CHO cells with TRPM1 and constitutively active Gα_o_ rendered the TRPM1 channels inactive^12^. Evidence indicating that Gβγ causes TRPM1 closure is based on results from several cell types, including bipolar cells, where dialyzing Gβγ reduced the mGluR6-initiated response, but dialyzing an activated form of Gα_o_ did not. Further support comes from transfected HEK cells and human melanocytes where Gβγ rather than active Gα_o_ reduced a Ca^2+^ signal triggered with high extracellular Ca^2+^ 22. These contradicting data could result from the use of different cell lines that express different endogenous molecules that impact the channel. Indeed, activating endogenous mGluR6 in melanocytes opens the channel instead of closing it; but after expressing Gα_o_ by transfection, mGluR6 activation closes the channel^23^. To better understand how the TRPM1 channel operates in retinal ON bipolar cells, we carried out a battery of experiments in which we dialyzed reagents that modify the status of the endogenous G-protein in rod bipolar cells and observed their effects on the basal current and on the light response. We further examined the interaction of G-protein subunits with TRPM1 using co-immunoprecipitation and energy transfer assays. Our results suggest that both Gα_o_ and Gβγ bind the TRPM1 channels and together cooperate to close them.

## Results

Our experiments involved blocking K^+^ and Cl^−^ channels, then dialyzing reagents into rod bipolar cells clamped at −60 mV, and then testing over time the reagents’ effects on the holding inward current. Given that most channels are blocked and only TRPM1 is likely to be gated by G_o_, the change in holding current indicates whether the TRPM1 channels are opening or closing in response to the dialyzed agent. We chose to monitor the holding current as the read out of the reagents’ effects rather than the size of the mGluR6-initiated response (as done by Shen *et al*.) because the mGluR6 response is likely to decrease regardless of whether channels are opened or closed by the reagent.

### In light-exposed retinas, dialyzing active Gα_o_ closes the TRPM1 channel

**Figure 1:**
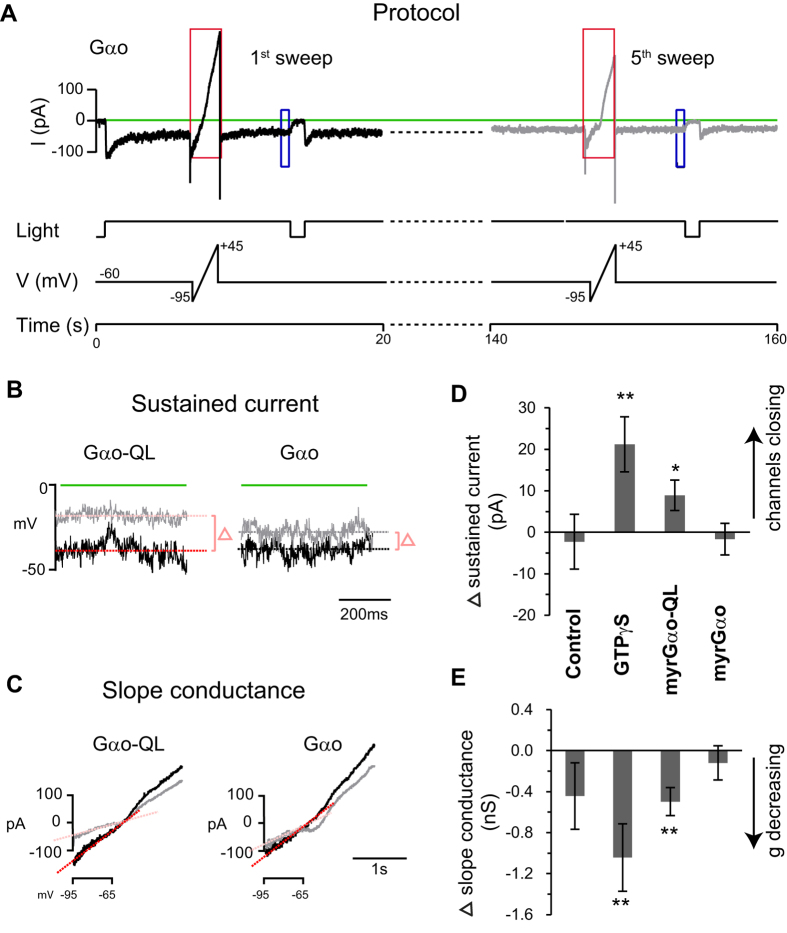
GTPγS and active myrGα_o_ close the TRPM1 channel (under light adaptation). (**A**) The protocol for light adapted conditions (illustrated with a record from a cell dialyzed with myr-Gα_o_): Immediately after break-in, cells were clamped at −60 mV, and then after 0.5 sec, a background light of 1.1 × 10^4^ photons μm^−2^ s^−1^ was applied; 6.5 s after break-in (and every 35 s thereafter), a 2 s voltage ramp from −95 mV to +45 mV was applied, and 13.5 s after break-in (and every 35 s thereafter), the light was turned off for 1 s (or occasionally for 4 s). Dotted lines between the 1^st^ (black) and 5^th^ (gray) sweeps indicate continued recordings. The initial light ON stimulus elicited a strong inward current that decayed due to adaptation. Blue and red boxes indicate the traces that are expanded in (**B,C**). (**B**) The sustained inward current under light was measured by averaging the currents over 0.5 sec before light OFF (indicated by blue rectangular boxes). The average inward current at the 1^st^ sweep (black trace) is drawn as a red line, and that at the 5^th^ (gray) is pink. The difference is indicated by the delta symbol. (**C**) Slope conductance was computed from the current responses to voltage ramp (shown in red boxes in **A**) between −95 mV to −65 mV (the linear range). The computed slopes are color coded as in (**B**). (**D**) Average differences in the sustained holding currents under light for four dialyzed solutions: control (normal pipette solution), 25 or 50 μM GTPγS, 40 nM constitutively active myrGα_o_-QL, or 100 nM WT myrGα_o_. When current doesn’t change, the difference is 0; when channels are closing, the inward current is less negative and the difference is positive. (**E**) Change in slope conductance for the same four solutions in (**D**). When the slope conductance decreases, the difference is negative. *indicates a significant difference (p < 0.05) between the 1^st^ and 5^th^ time points, and **indicates a highly significant difference (p < 0.01).

In order to test if Gα_o_ closes the channel, we had to first open the channels. Therefore, as soon as we established a seal, we provided a light step and then tested if the dialyzed solution closed the channel (see example in Fig. 1A). Every 35 sec we provided a voltage ramp to test slope conductance, and then briefly turned off the light to monitor OFF responses. Each of these 35-sec-protocol repetitions is referred to as a sweep, and a typical experiment had 5 sweeps. In general, the light step produced a large transient increase of the inward current followed by a smaller sustained current (Fig. 1A). Because our experiments required recording periods longer than the diffusion time (time scale of sec), and because BAPTA does not prevent the adapting fall off of the light response (as it only affects the response in the msec range), it was necessary to compare the sustained current that was achieved 13 sec after break-in. To compare this light-evoked sustained current before and after dialysis, we averaged the currents over 0.5 sec at the 1^st^ and 5^th^ sweeps (Fig. 1B). To determine if the dialyzed solution caused a statistically significant change, we applied the paired Students t-test. This test computes the probability that the average difference between the two time points is equal to 0 (i.e., it computes the probability that the dialyzed agent has no effect). We also provided voltage ramps to monitor changes in the slope conductance; this was computed in the linear range between −95 mV and −65 mV (to avoid contributions from the voltage-activated L-type calcium channel) (Fig. 1C). In control experiments (with only basic pipette solution), the inward sustained current remained relatively stable at around −34 pA (n = 14 cells; Table 1). Then, to test our method, we confirmed established observations that dialyzing GTPγS closes TRPM1^24^,^25^. When 50 μM GTPγS was perfused, only 5 out of 13 cells gave a light response. Since cells without responses could not be tested for channel closure, we increased the yield by switching to 25 μM GTPγS which evoked light responses in 6 out of 7 cells. Combining results from both concentrations, we found that the sustained current started at −37.3 ± 7.1 pA, and as expected, greatly decreased during dialysis. At the 5^th^ time point, the holding sustained current was −16.1 ± 4.1 pA, significantly lower than that at the 1^st^ time point (p < 0.01 paired Student’s t-test; Table 1). This indicates that GTPγS closes the channels that were opened by light. To illustrate the net effect of each dialyzed agent, we plotted the average difference in the sustained currents between the 5^th^ and the 1^st^ time points. Positive values indicate channel closure because the inward current becomes less negative (Fig. 1D).

Next we tested the effect of dialyzing a constitutively active mutant of Gα_o_. Previous experiments testing this effect activated the Gα_o_ subunit with GTPγS or with GMP-P(NH)P^12^,^14^,^22^. These approaches may have led to an excess of the non-hydrolysable GTP analogue in the solution, making it unclear whether the observed effect was caused only by active Gα_o_ or also by activation of Gβγ. Therefore we took the approach of producing a constitutively active mutant of myristoylated Gα_o_ (myrGα_o_-QL) and incubating it with GTP prior to introducing it into the cells. This QL mutation prevents GTP from being hydrolyzed, thus profoundly shifting its conformation to the active state in the presence of GTP^26^,^27^. We found that after dialyzing 40 nM of myrGα_o_-QL (with GTP as in the control solution), the light-evoked sustained current significantly dropped from −34.3 ± 6.3 to −26.1 ± 4.4 pA (n = 17, p = 0.029) (Fig. 1D and Table 1).

We then dialyzed wild type myristoylated Gα_o_ (100 nM), also after incubating it with GTP. Unlike the QL mutant, WT myrGα_o_ hydrolyzes all bound GTP within about 1–2 minutes^28^ and hence is expected to be largely inactive in its GDP-bound form when delivered into the cells. For WT myrGα_o_, the sustained current remained stable throughout the experiment (ranging between −29 pA and −31 pA; n = 17), as it did with the control solution (Fig. 1D). If a decrease in sustained current indicates that channels are closing, these current changes should correlate with changes in the slope conductance. Indeed, increases in sustained current were highly correlated with increases in slope conductance (R = −0.92; the minus sign results from inward currents being assigned negative values).

To confirm that these conductance changes were due to TRPM1 modulation, in several experiments we extended the light OFF period to 4 sec and provided a voltage ramp during this period. We then subtracted for each sweep the I-V curve during light OFF from that during light ON, thus isolating the contribution of TRPM1 to the measured current at different voltages. At the first sweep, TRPM1 contribution was significant (supp. Fig. 1), but at the 5^th^ sweep it was practically null, indicating that TRPM1 was closed and that the main difference seen during light ON was due to TRPM1. Thus we conclude that the closure of TRPM1 observed when dialyzing myrGα_o_-QL is due to Gα_o_’s active state. While we cannot compare the effect of Gα_o_-QL to that of GTPγS because of the difference in concentrations, diffusion properties, and nature of the activity, it appears that the effect of active myrGα_o_ is smaller than that of GTPγS. If so, this smaller effect may be due to an additional effect by Gβγ (which is also activated by GTPγS).

### Sequestering Gβγ opens the TRPM1 channel in the dark

**Figure 2:**
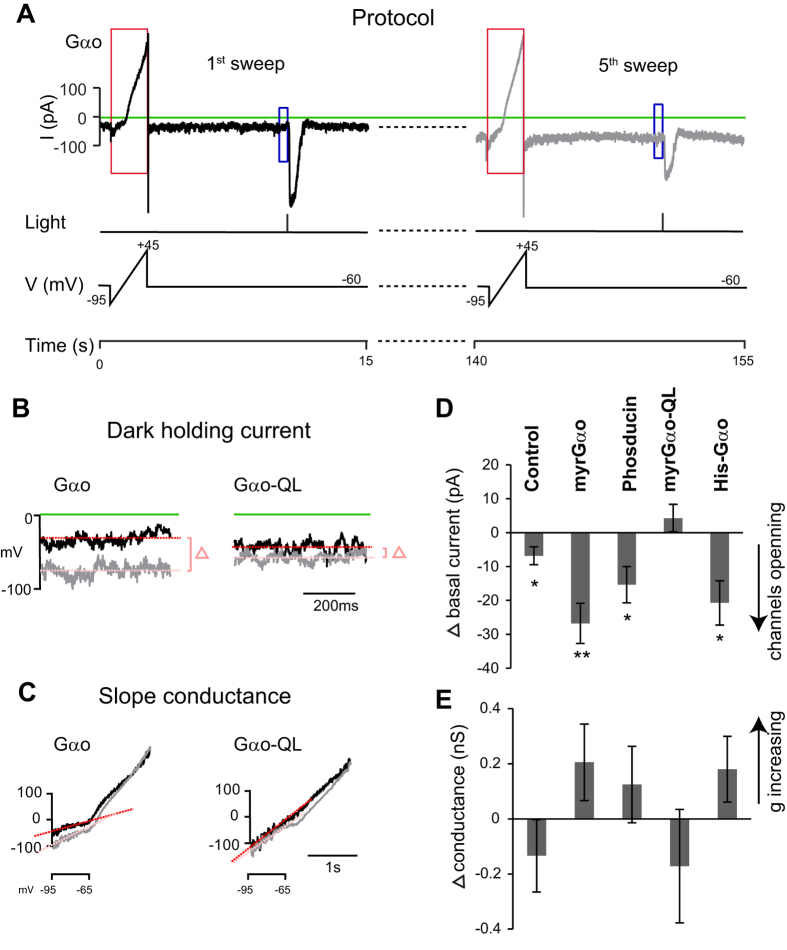
Sequestering Gβγ opens the TRPM1 channel (under dark adaptation). (**A**) The protocol of a dark adapted experiment (illustrated with a record from a cell dialyzed with myr-Gα_o_): 0.5 sec after break-in, a 2 s voltage ramp was applied, and 10.5 sec after break-in, a 50 msec light (3.8 × 10^4^ photons μm^−2^ s^−1^) was flashed. This cycle was repeated every 35 s. The 1^st^ sweep is shown in black, the 5^th^ sweep in gray. (**B**) The dark inward current was measured by averaging the currents over 0.5 sec before the light flash (indicated by blue rectangular boxes in A). Black trace is from the 1^st^ sweep, gray is from the 5^th^. The average holding current at the 1^st^ sweep is indicated by a red line, and that at the 5^th^ by a pink line. The difference is indicated by the delta symbol on the right. (**C**) Slope conductance was computed from the current responses to the voltage ramp (shown in red boxes in **A**) between −95 mV to −65 mV (the linear range). First ramp is in black, 5^th^ in gray. The slopes for these records are shown in red and pink, correspondingly. (**D**) Quantitative analysis of changes in the dark holding currents for five different conditions: control, 100 nM myrGα_o_, 9 μM phosducin, 40 nM myrGα_ο_−QL, and 150–300 nM His-Gα_o_. When channels are opening, the inward current increases (has a more negative value) and the difference between the 5^th^ and the 1^st^ time points is negative. (**E**) Quantitative analysis of changes in slope conductance for each of the above conditions. *indicates a significant difference (p < 0.05) between the 1^st^ and 5^th^ time points, and **indicates a highly significant difference (p < 0.01).

To test the effect of Gβγ on TRPM1, Shen *et al*. dialyzed recombinant Gβ1γ2 or native Gβγ subunits purified from the brain into rod bipolar cells^22^. They found that Gβγ reduced responses to light and to mGluR6 antagonists, suggesting that Gβγ closes the channel and prevents it from opening. In apparent disagreement, applying Gβγ to an inside-out excised patch of TRPM1-transfected CHO cells did not change the probability of channel opening^12^. To address these opposite findings, we tested the contribution of Gβγ in rod bipolar cells using the approach of inhibiting the endogenous Gβγ by sequestering it with Gα_o_-GDP or phosducin, a 28 kDa phospho-protein that binds Gβγ and thus inhibits activity of the free dimer^29^,^30^,^31^,^32^. These experiments were performed on dark-adapted cells to induce a state in which Gβγ is dissociated from the endogenous Gα_o_. The cells were clamped at −60 mV, and the dark holding current (which we term basal current) was measured at different time points after break-in (see example in Fig. 2A). Every 35 sec during the recording period, we provided a voltage ramp in darkness to test slope conductance followed by a strong 10 msec light flash to test the cell’s ability to produce a light response (Fig. 2A). For cells that were patched with control solution in the pipette, the average basal current at the first time point (10 sec after break-in) was −25.8 ± 4.1 pA, and that at the 5^th^ time point (150 sec after break-in) was −32.0 ± 3.2 pA (p = 0.06, n = 43 total over all dark experiments) (Fig. 2D and Table 2), indicating relative stability in the holding current.

Next, we dialyzed myrGα_o_ at both 40 nM and 100 nM concentrations. This increased the basal inward current in a concentration-dependent manner. While 40 nM myrGα_o_ increased the basal current at the 5^th^ time point by a factor of 1.22 (from −49.2 ± 7.5 pA to −60.1 ± 9.5 pA; n = 12; p = 0.4), 100 nM myrGα_o_ increased it by a factor of 2 (from −27.6 ± 3.6 pA to −54.4 ± 6.4 pA; n = 24; p < 0.01) (Fig. 2B,D). To determine if this increase in basal current is due to Gα_o_.GDP or if it would happen independent of Gα_o_’s nucleotide-bound state, we dialyzed myrGα_o_-QL (40 nM) and found it did not cause any change in basal current (37.2 ± 5.5 pA and −32.9 ± 4.1 pA for 1^st^ and 5^th^ time points, respectively; n = 22; p = 0.30) (Table 2 and Fig. 2D). We wondered if longer dialysis would make a difference, so for some experiments we measured the current at the 10^th^ time point. For myrGα_o_-QL, the holding current remained similar (from −38.4 ± 6.6 to −37.7 ± 5.3 pA; n = 16; p = 0.88) (sup Fig. 2), but for 40 nM WT myrGα_o_, the current continued to increase (−67.4 ± 10.9 at the 10^th^ time point; p = 0.03) (supp Fig. 2). These findings indicate that the opening of the TRPM1 channels by wild type Gα_o_ is due to its GDP-bound state.

Next, we tested the effect of dialyzing phosducin, a reagent that offers the advantage of preventing Gβγ from interacting with effectors without affecting the activation state of Gα and without changing its concentration^33^. When 9 μM phosducin was added to the pipette solution, the basal inward current progressively increased from −29.9 ± 5.9 pA to −45.3 ± 7.3 pA ; n = 14; p = 0.01) (Fig. 2D). This change in basal current highly correlated with the change in slope conductance: while this conductance decreased a little for control and myrGα_o_-QL, it increased for both WT myrGα_o_ and phosducin. The correlation between basal current and slope conductance was −0.91 (Fig. 2C,E), supporting the notion that an increase in basal current indicates channel opening. Thus, our results show that dialyzing reagents that sequester Gβγ opens TRPM1 channels, suggesting that Gβγ closes the channel.

### Linoleic and myristic acids do not modulate TRPM1

In the experiments above, we used myristoylated forms of Gα_o_ because native Gα_o_ harbors this post-translational modification that is important for its normal association with the membrane^34^. However, because lipids are well known to mediate or modulate gating of TRP channels^35^,^36^,^37^, we tested if the myristoyl group present on myrGα_o_ may contribute directly to channel opening in our experiments by using non-myristoylated Gα_o_ where the myristoylation signal at the N-terminus was replaced with a His_6_ affinity tag (His-Gα_o_). Similar to myrGα_o_, 150 or 300 nM His_6_-Gα_o_ increased the basal inward current (from −7.9 ± 2.7 pA to −28.7 ± 7.0 pA; n = 9; p = 0.013) as well as the slope conductance (Fig. 2D,E).

**Figure 3:**
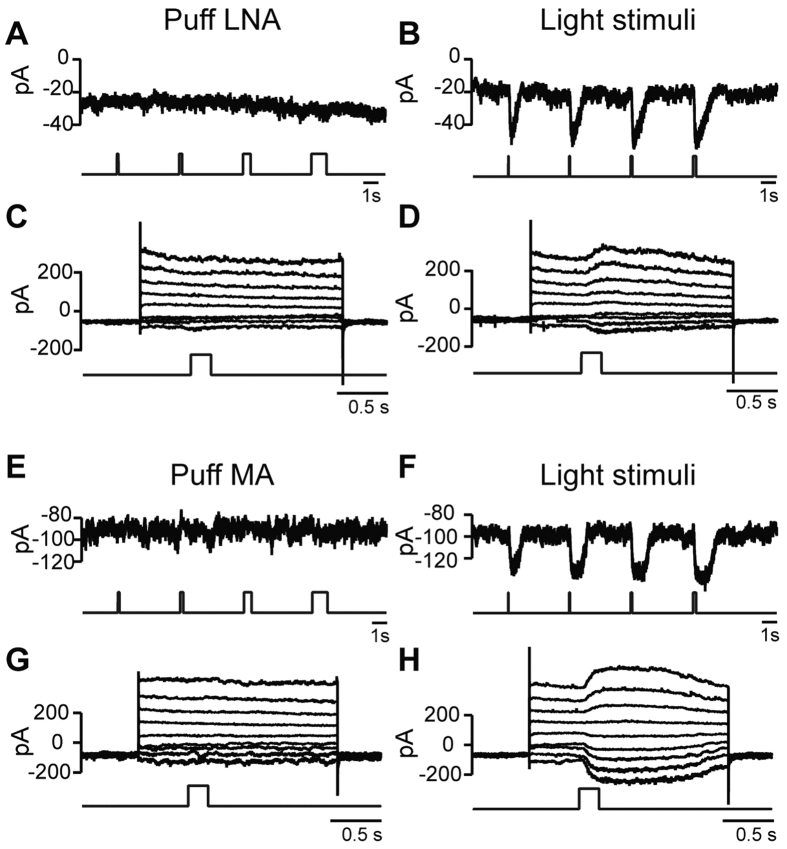
Alpha linoleic acid and myristic acid do not modulate the channel. (**A**) An example of a cell clamped at −60 mV and puffed with 100 μM alpha linoleic acid (LNA) of different puff durations indicated by the lower trace. (**B**) Same cell stimulated with light for different durations. (**C**,**D**) Same cell clamped at different voltages from −95mV to +65mV with 20 mV steps and either puffed with LNA for 0.2 sec (**C**) or flashed for 0.2 sec (**D**). The cell responds to light, but not to LNA. (**E**–**H**) As for (**A**–**D**), but puffed with 100 μM myristic acid (MA). Again, no response to the myristic acid was observed.

Next, since puffing certain lipid modifiers on the extracellular face of the plasma membrane can modulate certain TRP channels^38^,^39^,^40^, we further tested TRPM1 modulation by puffing alpha linoleic acid (LNA, 20–100 μM; 5 cells) or myristic acid (MA, 100–250 μM; 8 cells). We found that although these cells responded to light, they did not respond to the lipid modifiers (Fig. 3), indicating that the main mechanism for channel opening is solely through the G-protein.

### The TRPM1 channel stably associates with both Gα_o_ and Gβ3γ13

**Figure 4:**
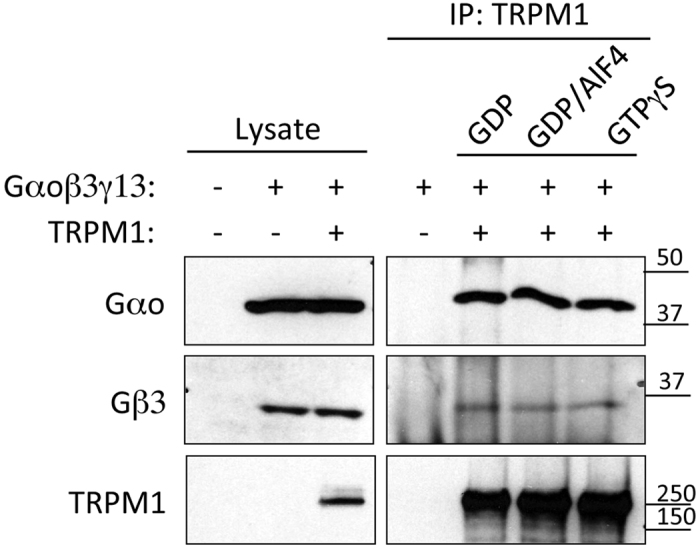
Gα_o_ and Gβ3γ13 co-immunoprecipitate with TRPM1. Interaction between TRPM1 and G-protein subunits was studied upon co-transfection into HEK293T cells. Cells were lysed and TRPM1 was precipitated by specific antibodies against TRPM1. Proteins present in the lysates and IP eluates were detected by Western blotting. Approximately 6-fold more material was loaded for the eluates relative to the amount of material present in the lysates. Quantification of protein content revealed 70–100% IP efficiency for TRPM1, 10–12% for Gα_o_ and 7–14% for Gβ3 across samples with no significant differences between nucleotide states. The shown Western blots were cropped at the expected molecular weight; both Gα_o_ and Gβ3γ13 were precipitated regardless of added nucleotides.

Our functional experiments suggest that both Gα and Gβγ subunits of G_o_ regulate the TRPM1 channel open state. To support this model with biochemical evidence and to determine if the effect is direct, we examined the interaction of the G-protein subunits with the TRPM1 channel. First, we co-expressed TRPM1 in HEK293T cells with Gα_o_ and the ON bipolar-specific Gβγ combination Gβ3γ13. Following cell lysis, TRPM1 was immunoprecipitated and examined for its ability to pull-down Gα_o_ under three conditions: **(1)** in Gα_o_’s basal state when the lysates were incubated with GDP; **(2)** in Gα_o_’s transition state induced by AlF4; and **(3)** in Gα_o_’s activated state in the presence of GTPγS. We found that anti-TRPM1 effectively co-immunoprecipitated Gα_o_ as well as Gβ3γ13 (3 experiments; Fig. 4). This binding was equivalent across all conditions, suggesting that the association of TRPM1 with Gα_o_ is independent of the activity state of Gα_o_. No binding was observed in the absence of TRPM1, indicating that this interaction is specific.

**Figure 5:**
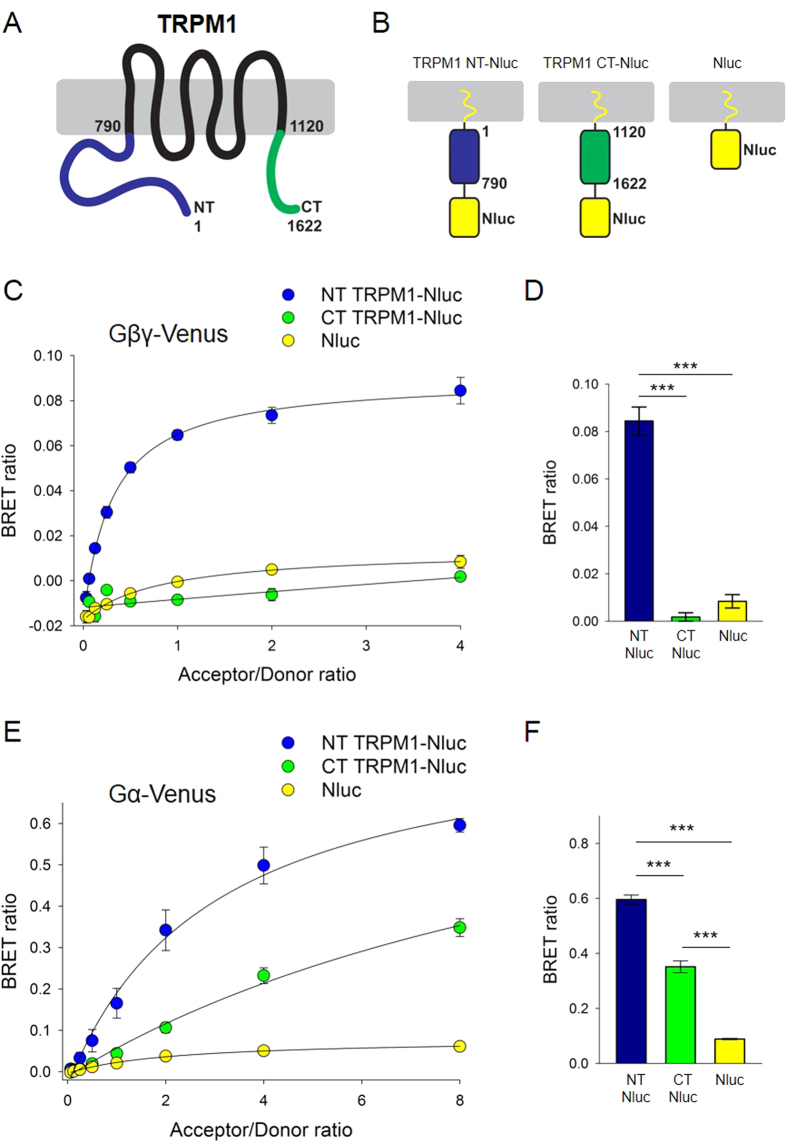
BRET analysis shows interaction between TRPM1-NT and TRPM1-CT with Gα_o_ and Gβγ. HEK293T cells were transfected with fixed amounts of cDNAs coding for TRPM1-derived proteins fused to Nanoluc (donor) and increasing concentrations of cDNAs coding for Gα_o_ or Gβ1γ2 fused to Venus (acceptor). (**A**) Scheme of TRPM1 with amino acid references of N-terminus (blue) and C-terminus (green). (**B**) Schemes of the TRPM1-derived constructs used as donors. Each construct had a membrane localization signal at the N-terminus (yellow squiggle) and Nanoluc (Nluc) at the C-terminus. (**C**) BRET titration assays were performed by measuring energy transfer with increasing concentrations of Gβ1γ2-Venus and fixed amounts of TRPM1-NT-Nluc or TRPM1-CT-Nluc (mean ± S.E.; n = 3 experiments from 3 different transfections).(**D**) Quantification and statistical analyses of the BRET assay reported in panel C at the saturating condition of 4:1 acceptor/donor ratio (mean ± S.E.; n = 3; ***p < 0.001; one way ANOVA followed by Bonferroni post-hoc test). (**E**) BRET titration assays with increasing concentrations of Gα_o_-Venus and fixed amounts of TRPM1-NT-Nluc or TRPM1-CT-Nluc (mean ± S.E.; n = 3). (**F**) Quantification and statistical analyses of the BRET assay reported in panel E at the 8:1 acceptor/donor ratio (mean ± S.E.; n = 3; ***p < 0.001; one way ANOVA followed by Bonferroni post-hoc test).

We further studied the association of G_o_ subunits with the TRPM1 channel using a Bioluminescence Resonance Energy Transfer (BRET) assay. In this approach, cytoplasmic N-terminal and C-terminal domains of TRPM1 were fused with a highly efficient energy donor (Nluc) and paired with the Gβγ or Gα_o_ subunits fused with Venus, the fluorescent acceptor (Fig. 5A,B). In these experiments we used a prototypic Gβγ pair, Gβ1γ2, for its functional equivalence and effectiveness in gating the TRPM1 Channel^22^. To direct the TRPM1 fragments to the plasma membrane where the G-protein subunits are naturally found, the constructs were further appended with an engineered membrane localization sequence. When Gβγ was co-transfected with the N-terminus of TRPM1, the acceptor/donor titration experiments revealed a hyperbolic profile of the BRET signal that saturated at an acceptor/donor ratio of about 1 (Fig. 5C). In contrast, when Gβ1γ2 was combined with the C-terminus of TRPM1, the BRET signal was not different from the shallow linear signal observed with membrane-targeted Nluc luciferase (Fig. 5C,D). This suggests that under our experimental conditions only the N-terminus of TRPM1 specifically interacts with Gβγ.

When Gα_o_ was used as an energy acceptor, both the N-terminus and the C-terminus fragments produced significant BRET signals, but the N-terminus gave a stronger signal (Fig. 5E,F). These results indicate that both Gα_ο_ and Gβγ interact with the TRPM1 channel, and they further localize the site of interaction: while Gα_o_ interacts with both ends of TRPM1, Gβγ appears to interact only with the N-terminus.

## Discussion

We present evidence that both Gα_o_ and free Gβγ play a role in modulating the TRPM1 channel open-state. Furthermore, the close association of these subunits with TRPM1 suggests that the actions of the G-protein subunits are direct rather than acting via a second messenger. To our knowledge, this is the first example of a TRP channel that is directly gated by both arms of a G-protein. TRPC4 has been shown to interact with Gα_i2_, but not with Gβγ^41^.

### Role of Gα_o_

The critical piece of evidence that supports the role of Gα_o_ is our finding that dialyzing a constitutively active mutant of this subunit during light exposure leads to channel closure. Under a prolonged strong light stimulus, the majority of the G-protein must be in its inactive form where Gβγ is bound to Gα_o_.GDP. Because the dialyzed Gα_o_-QL does not affect Gβγ, the observed channel closure is attributed to the dialyzed reagent and not to Gβγ. Since dialyzing wild type Gα_o_ did not change the channel open-state, we conclude that Gα_o._GTP contributes to channel closure. This conclusion agrees with experiments showing that application of GMP-P(NH)P-activated Gα_o_ to an excised patch of TRPM1-transfected CHO cells closes the channel, and that transfection with Gα_o_Q205L renders the channel closed^14^. The idea that active Gα_o_ contributes to channel modulation is further supported by our findings that in HEK cells, Gα_o_.GTP physically associates with TRPM1. Based on these experiments and the excise patch experiment^14^, we suggest that the action of Gα_o_ is direct.

### TRPM1 open-state requires associated proteins

TRPM1 is thought to be constitutively open because in ON bipolar cells its closure requires activation of G_o_. If so, in situations when G_o_ is naturally inactive, such as in rod bipolar cells lacking mGluR6, TRPM1 channels are expected to stay open. Contrary to this expectation, we previously found that the resting membrane potential in rod bipolar cells lacking mGluR6 is more hyperpolarized than in WT cells by about −15 mV, and the holding current is similar to that of *TRPM1*-KO^14^,^42^. This suggests that TRPM1 requires additional components to stay open. This requirement for an associated protein has also been proposed in two other studies. In the first, it was suggested because TRPM1 was shown to lack 4-fold symmetry^43^, characteristic of channel oligomerization seen for TRPV1 and other TRP channels, and in the second becasue capsaisin could not stimulate heterogeneously expressed TRPM1^44^. One possibility is that Gα_o_ is this auxiliary protein, and four lines of evidence support this idea. **(1)** In melanocytes, which natively do not express Gα_o_, TRPM1 appears to be constitutively closed and stimulation of mGluR6 opens the channel. When the cells are transfected with Gα_o_, activation of mGluR6 closes the channel^23^. **(2)** In rod bipolar cells lacking Gα_o1_, TRPM1 seems closed^8^ even though the channel is expressed in the dendritic tips as in WT cells, and Gβγ is practically absent^45^. **(3)** Gα_o_.GDP interacts with TRPM1 (this study). **(4)** Dialyzing Gα_o_.GDP is extremely efficient in opening the channel (this study), suggesting that some of the observed effect can be due to direct interaction and not only to sequestering Gβγ. While Gα_o_ may be an auxiliary protein, it is unlikely the only one that support channel opening since ON bipolar cells lacking mGluR6 still express Gα_o_^42^,^46^ yet the TRPM1 channels are closed. At least two proteins are required for stable expression of TRPM1 in the membrane, nyctalopin and LRIT3, and in their absence rod bipolar cells are unresponsive to light and the channel is closed or absent from the dendritic tips^47^,^48^. Thus it is possible that these two proteins, mGluR6, and/or other unknown components contribute not only to trafficking and stable expression, but also to maintaining open TRPM1 conformations.

### Cooperation between Gα and Gβγ

**Figure 6:**
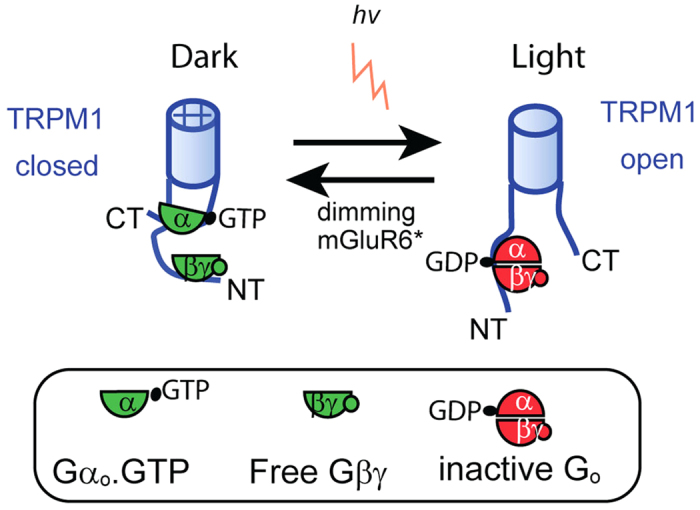
A model for TRPM1 gating. In the dark, mGluR6 is bound to glutamate and G_o_ is active (depicted in green). Both active Gα_o_ and free Gβγ contribute to channel closure, and both can interact with TRPM1. We speculate that Gβγ is bound to the N-terminus and Gαo.GTP to the C-terminus; both twisting the channel to close it. When light decreases glutamate, and hence deactivates G_o_ (depicted as red), the heterotrimer G_o_ is reformed and allows the channel to open. Not shown in the model are other proteins that must interact with this complex to allow these activities to cycle; in particular, mGluR6 must bind the inactive G_o_, and the GTPase-activating complex must bind Gα_o_.GTP.

While the classical view of G-protein function is that upon GTP/GDP exchange, Gα.GTP activates its effector, there are several examples where the effector is activated by Gβγ^49^,^50^. Interestingly, most known Gβγ effectors, including adenylyl cyclase, PLCβ, and the G-protein-gated inwardly rectifying potassium channel (GIRK), are also effectors for Gα; i.e., Gα cooperates with Gβγ to modulate the downstream activity^51^,^52^,^53^,^54^,^55^. The GIRK channel provides an especially interesting example because being a channel, the function and interaction of the G-protein subunits with it can readily be compared to their function and interaction with TRPM1. It is well known that GIRK is directly gated by Gβγ^56^,^57^, but the function of Gα_i/o_ in the GIRK-Gαβγ complex is emerging more slowly. It is now understood that the non-activated Gα_i3_ has 3 independent functions: it reduces the basal current of GIRK, enhances the evoked current, and regulates its kinetics^58^,^59^,^60^. Both Gα_i3_.GDP and Gα_i3_.GTP interact and regulate GIRK1/2^61^. Thus, the analogy of the interaction of the G-protein with the GIRK channel to that with TRPM1 holds on several levels, but the effects of the G-protein subunits are opposite. While Gβγ opens GIRK, it closes TRPM1. While Gα_i/o_.GDP reduces the basal GIRK current, Gα_o_.GDP seems to increase the basal TRPM1 current. In either case, both Gα.GDP and Gα.GTP are retained in the complex, and Gα.GTP works synergistically with Gβγ 61. The simplest model that can explain the TRPM1-G-protein interaction (summarized in Fig. 6) is that Gα_o_.GDPβγ binds TRPM1 and endows it with an open conformation; when GTP replaces GDP, Gβ3γ13 dissociates from Gα_o_.GTP and both arms twist TRPM1 and change its conformation to the close state. Gβγ binds to TRPM1 probably via its N-terminus, while Gα_o_ may bind both ends of TRPM1. We speculate that Gα_o_ swings from one terminus to the other upon nucleotide exchange; when GDP-bound, it joins Gβγ at the N-terminus, and when GTP-bound, it binds the C-terminus. It is important to remember that the macromolecular complex must contain other proteins as well since Gα_o_.GDPβγ must also bind mGluR6 to be activated, and Gα_o_.GTP must also bind the GAP complex to be deactivated.

## Materials and Methods

### Ethical approval

Procedures involving animals were performed in accordance with National Institute of Health guidelines and the protocol was reviewed and approved by the Institutional Animal Care and Use Committee of the University of Pennsylvania and the competent ethics committees at Jinan University. C57BL/6J wild type mice (WT) were purchased from Charles River laboratories. A mouse was deeply anesthetized by intraperitoneal injection of a mixture of 100 μg/gm ketamine and 10 μg/gm xylazine; the eyes were enucleated and the mouse was euthanized by anesthetic overdose.

### Whole cell recording experiments

#### Recording

Retinal slices were prepared as described previously^62^. Briefly, retinas were isolated under red light and cut into 200 μm thick slices with a tissue slicer (Narishige, Japan). The slices were transferred to a recording chamber, secured with vacuum grease and then moved to the microscope stage of an Olympus microscope equipped with a 60x water immersion objective. The chamber was perfused at a rate of 0.5–1 ml/min with oxygenated (95% O_2_, 5%CO_2_) Ames medium (Sigma, St. Louis, MO) containing sodium bicarbonate (1.9 g/l) and 2 μM Strychnine and 100 μM picrotoxin (to block GABA_A/C_ receptors) at 32–34°C.

Patch pipettes with resistances of 7–9 MΩ were fabricated from borosilicate glass using an electrode puller (Sutter, Novato, CA). Pipettes were filled with the following solutions (in mM): 108 Cs-gluconate, 10 BAPTA, 10 HEPES, 10 NaCl, 4 MgATP and 1 LiGTP. The pH was adjusted to 7.4 with KOH and the osmolality was 290 mOsmol. All chemicals were obtained from Sigma. The solution was aliquoted, stored at –20 °C and thawed before each experiment. For each set of experiments, we patched several cells with electrodes that contained the above control solution, and several with electrodes that also contained a modifier reagent as explained in Results.

#### Source of modifier reagents

Myristoylated Gα_o1_ (hence referred to as myrGα_o_) was obtained from Calbiochem and used for most experiments; myrGα_o_-Q205L and its control myrGα_o_ were prepared as described below; GTPγS was obtained from Sigma (Sigma-Aldrich, St Louis, MO); phosducin was a gift from Dr. Vadim Arshavsky (Duke University); and His_6_-Gα_o_ was a gift from Dr. Richard Neubig (University of Michigan).

Current recordings in the US were obtained with an Axopatch 1D amplifier (Molecular Devices) and in China with an EPC−10 patch clamp amplifier (HEKA, Lambrecht, Germany). Membrane potentials were corrected for liquid junction potential calculated to be ~15 mV. Cells were discarded if the baseline current in the dark exceeded −100 pA at a holding potential of −60 mV. Voltage command generation and data acquisition were accomplished with Clampex (Molecular Devices) or PatchMaster (HEKA). Cells were voltage clamped at −60 mV and the holding current and light-evoked current responses were compared over time for control and different dialyzed reagents. It has been reported that clamping a cell at +50 mV may extend the recording time because less calcium-dependent desensitization occurs^63^. However, in our hands, such clamping did not prove beneficial, and we preferred to make the measurements under more physiologically-relevant voltages.

#### Light stimuli

The retina was stimulated using a green full-field light generated by a light emitting diode with a peak wavelength of 565 nm (or 500 nm in China). During dark adaptation, the light response was tested with a 10 ms flash (3.8 × 10^4^ photons/μm^2^/s) that was turned on every 35 s. To measure changes in slope conductance, a voltage ramp from −95 mV to +45 mV was applied every 35 s. During light adaptation, a background illumination with an intensity of 1.1 × 10^4^ photons/μm^2^/s was applied immediately after break-in. To test the OFF response, the light was turned off for 1 or 4 s every 35 s. Slope conductance was measured every 35 s while the light was ON. In some cases another ramp was applied during light OFF.

#### Analysis

For each cell, the baseline current was calculated every 35 s as the average of current for 0.5 s before light ON for dark-adapted retinas or light OFF for light- adapted retinas. The light response was measured as the peak response to a flash of light (ON response). The slope conductance was measured from the linear range between −95 mV to −65 mV. Waveform analysis of the response was done off-line with Clampfit (Molecular Devices). All data are reported as mean ± SEM (Standard Error of the Mean). The values of the holding current and conductance at different time points after break-in were compared to those at the first sweep using paired Student’s t-test using Excell. Differences were considered significant when p ≤ 0.05. For p values above 0.01, we report the actual value, and for values below 0.01, we simply state p < 0.01.

### Expression and purification of the myristoylated forms of rat Gα_o1_ and Gα_o1_−Q205L subunits (hence referred to as myrGα_o_ and myrGα_o_−QL)

For Gα_o_ bacterial expression vector, the full-length rat Gα_o1_ (or Gα_o1_-Q205L mutant) cDNA was fused to a C-terminal His_6_ tag and cloned into pET21 (Novagen). pHV738 (a gift from Dr.Richard Kahn, Emory University), a vector which contains the human N-myristoyltransferase 1 (hNMT1) and E. coli methionine aminopeptidase (map) genes, was used to express the eukaryotic N-myristoylation machinery^64^. To increase N-myristoylation efficiency, the E. coli formylmethionine deformylase (def) gene additionally was inserted in pHV738, and the resulting plasmid was called pHV738/def. The E. coli strain BL21-CodonPlus(DE3)-RIPL (Stratagene) was simultaneously transformed with pET21-Gα_o_WT/His_6_ (or pET21-Gα_o_-QL/His_6_) and pHV738/def. The transformed cells were grown in terrific broth (TB) supplemented with ampicillin (50 mg/ml), kanamycin (25 mg/ml), and chloramphenicol (34 mg/ml). Bacteria were inoculated and allowed to grow at 37 °C; when the culture reached OD_600_ of 0.6, the temperature was reduced to 25 °C. At OD_600_ of 0.8, 50 μM sodium myristate (Sigma # M8005) was added, and 10 min later induction of gene expression was triggered with 1M isopropyl-β-D-thiogalactopyranoside (IPTG) (Invitrogen). Cells were incubated under gentle agitation and harvested 16 hours later by centrifugation. Cell pellets were resuspended and sonicated on ice for 4 min (5 sec on/15 sec off). The crude lysate was clarified by centrifugation and purified (at 4 °C) by chromatography over Ni-NTA. MyrGα_o_ was eluted with 10 mM EDTA in 50 mM Tris-HCl, pH 7.5. The eluted protein fractions were collected and analyzed by SDS-PAGE. Fractions containing myrGα_o_ were pooled, concentrated, and further purified on a Superdex 200 column (GE Healthcare). The purity of myrGα_o_ was confirmed by SDS-PAGE gel analysis. After purification, the biological activity of recombinant Gα_o_ subunits was tested by [^35^S]GTPγS binding. On average, 1 mole of myrGα_o_ bound 0.4–0.6 moles of GTPγS. N-myristoylation, determined by mass spectrometry, showed 80–85% of the protein to be myristoylated. The protein was stored at −80 °C. Further details about purification and testing of the material will be published elsewhere.

### DNA constructs

Plasmids used for co-IP experiments from transfected cells were generated as follows: mouse TRPM1 full-length (NM_001039104.2transcript variant 2) in pcDNA3.1 was previously described^65^; human Gβ3 (NM_002074.4 transcript variant 1) and human Gγ13 (NM_016541.2) in pcDNA3.1 were purchased from cDNA.org (cat# GNB0300000–02 and GNG1300000-02); rat Gα_o1_ (NM_017327). Plasmids encoding Bioluminescence Resonance Energy Transfer (BRET) sensors were generated using In-Fusion HD cloning system (Clontech) in pcDNA3.1. As templates, we used plasmids encoding the mouse TRPM1 full-length in pcDNA3.1^65^ and the Nanoluc-encoding plasmid pNL1.1 (Promega). Nanoluc was fused in-frame at the C-terminus of the sequences of TRPM1-NT (aa 1-790; TRPM1-NT-Nluc) or TRPM1-CT (aa 1120-1622; TRPM1-CT-Nluc) in pcDNA3.1. The myristoylation signal was added to the N-terminus of each construct to facilitate membrane localization of respective proteins. BRET sensor constructs Venus155–239-Gβ1 (human; NM_002074.4 transcript variant 1. Venus sequence corresponding to aa 156-239 was added in the N terminus of Gβ1 separated by the linker sequence GGSGGG), Venus1–155-Gγ2 (human; NM_053064.4 transcript variant 1. Venus sequence corresponding to aa 1-155 was added in the N terminus of Gγ2 separated by the linker sequence GGSGGG) and Venus-Gα_o1_ (human; NM_020988.2 transcript variant 1. Venus sequence was inserted in Gα_o_ sequence between aa 91-91 flanked on each side by 4 glycine residues) were provided by N.A. Lambert (Medical College of Georgia, Augusta, GA). All constructs were verified by sequencing.

### Co-immunoprecipitation Assays

HEK293T cells were grown in six-well plates and transfected with Lipofectamine LTX (Invitrogen). Transfected plasmids encoded the following constructs (per well): 0.42 μg Gα_o_, 0.42 μg Gβ3, 0.42 μg Gγ13 and 1.25 μg TRPM1 or 1.25 μg empty pcDNA3.1 vector. After 24 hours, cells were harvested and lysed by sonication in ice-cold PBS IP buffer (150 mM NaCl, 1% Triton X-100, 5 mM MgCl_2_ and Complete protease inhibitor tablets) supplemented with three different compositions (GDP: 0.01 μM GDP; GDP/AlF_4_: 0.01 μM GDP, 10 mM NaF and 0.02 mM AlCl_3_; GTPγS: 0.01 mM GTPγs). Lysates were cleared by centrifugation at 14,000 rpm for 10 minutes. The supernatant was incubated with 20 μl of 50% protein G slurry (GE Healthcare) and 3 μg sheep anti-TRPM1 antibody on a rocker at room temperature for 1 hour. After three washes with the indicated IP buffer, proteins were eluted from beads with 50 μl of 2X SDS sample buffer. Proteins retained by the beads were analyzed with SDS-PAGE, followed by Western blotting using HRP conjugated secondary antibodies and an ECL West Pico (Thermo Scientific) detection system. Signals were captured on film and scanned by densitometer.

### BRET experiments

HEK293T/17 cells were cultured at 37 °C and 5% CO_2_ in DMEM supplemented with 10% fetal bovine serum, MEM non-essential amino acids and 1 mM sodium pyruvate. Cells were plated at a density of 50,000 cells/well in a white 96 well plate with clear bottom (Greiner Bio-One) and transfected using Lipofectamine LTX (Invitrogen) and PLUS^TM^ Reagent (Invitrogen). Cells were co-transfected with a fixed concentration of Nanoluc-fused constructs (donors) and increasing concentrations of Venus-fused constructs (acceptors). Empty vector was used to balance the amount of transfected DNA. Readings were obtained 24 h after transfection, immediately following media exchange to PBS containing 0.5 mM MgCl_2_ and 0.1% glucose and Nanoluc substrate (Nano-Glo, Promega) diluted 1:100. Fluorescence (Venus; 535 nm with 30 nm band path width) and luminescence (Nanoluc; 475 nm with 30 nm band path width) emissions were recorded simultaneously in real time with a microplate reader (POLARstar Omega, BMG Labtech) equipped with two photomultiplier tubes. The BRET signal was calculated as the ratio of the light emitted by acceptor over the light emitted by the donor. The ratio of emissions at acceptor and donor wavelengths from donor-only samples has been subtracted. All measurements were performed at room temperature.

## Additional Information

**How to cite this article**: Xu, Y. *et al*. The TRPM1 channel in ON-bipolar cells is gated by both the α and the βγ subunits of the G-protein G_o_. *Sci. Rep.* **6**, 20940; doi: 10.1038/srep20940 (2016).

## Supplementary Material

Supplementary Information

## Figures and Tables

**Table 1:** Summary of light-evoked sustained currents under different dialyzed solutions.

	**Control**	**GTPγS**	**myrGαo**	**myrGαo-QL**
I (1^st^) ± SEM	−33.80 ± 8.25	−37.27 ± 7.09	−29.25 ± 4.91	−34.31 ± 6.26
P (reagent vs control at 1^st^)		0.77	0.64	0.96
I (5^th^) ± SEM	−36.07 ± 6.51	−16.07 ± 4.10	−30.92 ± 4.11	−26.15 ± 4.37
P (reagent vs control at 5^th^)		0.03	0.51	0.21
P (paired Student t-test)	0.74	0.009	0.67	0.029

The first row shows the light-evoked sustained currents (I) ± SEM for the first time point; the second row shows the P values obtained with Student’s t-test when comparing the data for each reagent at the 1^st^ time point to that of control at the 1^st^ time point. The third row shows the currents (I) ± SEM for the 5^th^ data point and the fourth row shows the P value obtained with Student’s t-test when comparing the data for each reagent at the 5^st^ time point to that of control at the 5^st^ time point. The last row shows the P values obtained with paired student t-test comparing 1^st^ to 5^th^ time points for each reagent.

**Table 2:** Summary of basal currents in the dark under different dialyzed solutions.

	**Control**	**myrGαo**	**His Gαo**	**phosducin**	**myrGαo-QL**
I (1^st^) ± SEM	−25.8 ± 4.1	−27.6 ± 3.6	−7.9 ± 2.7	−29.9 ± 5.9	−37.2 ± 5.5
P (reagent vs control at 1^st^)		0.765	0.054	0.595	0.103
I (5^th^) ± SEM	−32.0 ± 3.2	−54.4 ± 6.4	−28.7 ± 7.0	−45.3 ± 7.3	−32.9 ± 4.1
P (reagent vs control at 5^th^)		0.0009	0.668	0.061	0.861
P (paired Student t-test)	0.062	0.0002	0.013	0.013	0.301

For explanation refer to Table 1, except that here we present the basal current under dark.
