# Supplementary material for: The TRPM1 channel in ON-bipolar cells is gated by both the α and the βγ subunits of the G-protein Go
Source: Sci Rep. 2016 Feb 17;6:20940. doi: 10.1038/srep20940 (PMC4756708; doi:10.1038/srep20940)
Supplement: Supplementary Information [file srep20940-s1.pdf]

The TRPM1 channel in ON-bipolar cells is gated by both the α and the βγ subunits of the G-protein G_o

Ying Xu(徐颖)[1,5], Cesare Orlandi[2], Yan Cao[2], Shengyan Yang（杨昇炎）[1], Chan-Il Choi[3], Vijayakanth Pagadala[3], Lutz Birnbaumer[3], Kirill A. Martemyanov[2], Noga Vardi[4]

Supplementary Figures

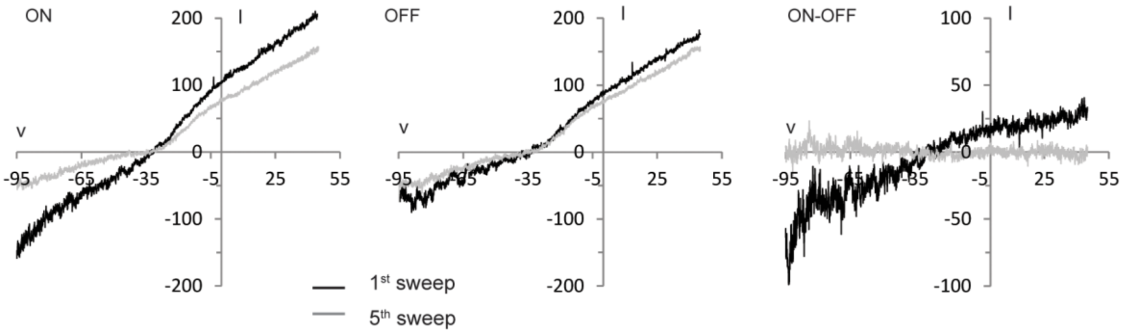

Supplementary figure 1. Only TRPM1 is closed by myr$G\alpha_o$-QL. An example of I-V curves taken from a cell dialyzed with $G\alpha_o$-QL for the 1st and 5th sweeps during light ON (left) and during light OFF (middle). The difference between the curves during light ON and OFF (right) gives the contribution of TRPM1. Note that at the 1st sweep (black), TRPM1 has a significant conductance while at the 5th sweep (gray), the curve is flat, indicating TRPM1 is closed.

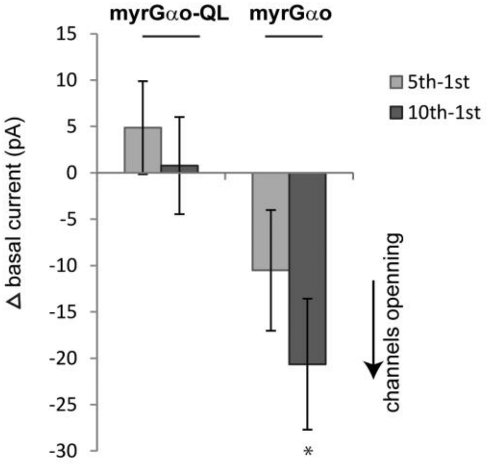

Supplementary figure 2. Under dark-adaptation, 40 nM myr$G\alpha_o$, but not 40 nM myr$G\alpha_o$-QL, increases the holding current. Plotted are the differences between the basal currents at the 5th time point minus that in the first (light gray) and that between the 10th time point and the 1st time point (darker gray) for WT myr$G\alpha_o$ (right bars) and the mutated active form (left bars). When channels are opening, the inward current increases (has a more negative value) giving negative values. For WT the difference increased with time, but for QL it remained practicably the same.
